# 

**Published:** 1894-09

**Authors:** 


### CONTENTS FOR SEPTEMBER, 1894.

ORIGINAL COMMUNICATIONS. PAGE.

On the Prognosis and Treatment of Proliferous Inflammation of the Middle Ear. By LUCIEN HOWE, M. R. C. S., Eng.......... 65
Some Affections of the Eye Associated With and Dependent Upon the Scrofulous Diathesis. By ALVIN A. HUBBELL, M. D.......... 76

SOCIETY PROCEEDINGS.

Louisville Surgical Society.......... 85
Medical Society of the County of Erie.......... 92

PROGRESS IN MEDICAL SCIENCE.

Ophthalmology. By ALVIN A. HUBBELL, M. D.......... 96
Neurology. By WILLIAM C. KRAUSS, M. D.......... 104

SELECTIONS.

An Easy Method of Bathing in Typhoid Fever.......... 106
The Conduct of Ordinary Labor Through External Examinations Solely.......... 108
Extra-Genital Syphilitic Infection.......... 109

CORRESPONDENCE.

Philadelphia Letter.......... 111

EDITORIAL.

Summer Intestinal Diseases of Children.......... 114
Topics of the Month.......... 115

---

Personal.......... 120
Society Meetings.......... 121
Medical College Notes.......... 122
Book Reviews.......... 124
Literary Notes.......... 127

